# Distribution of CRISPR Types in Fluoroquinolone-Resistant *Campylobacter jejuni* Isolates

**DOI:** 10.3390/pathogens10030345

**Published:** 2021-03-16

**Authors:** Mehmet Cemal Adiguzel, Debora Brito Goulart, Zuowei Wu, Jinji Pang, Seyda Cengiz, Qijing Zhang, Orhan Sahin

**Affiliations:** 1 Department of Microbiology, College of Veterinary Medicine, Ataturk University, Erzurum 25240, Turkey; mcemal.adiguzel@atauni.edu.tr (M.C.A.); seydacengiz@atauni.edu.tr (S.C.); 2 Department of Veterinary Microbiology and Preventive Medicine, College of Veterinary Medicine, Iowa State University, Ames, IA 50011, USA; dgoulart@iastate.edu (D.B.G.); wuzw@iastate.edu (Z.W.); pjj0702@iastate.edu (J.P.); zhang123@iastate.edu (Q.Z.); 3 Department of Veterinary Diagnostic and Production Animal Medicine, College of Veterinary Medicine, Iowa State University, Ames, IA 50011, USA

**Keywords:** *Campylobacter jejuni*, *Cas9* gene, CRISPR-Cas system, fluoroquinolone-resistant bacteria

## Abstract

To aid development of phage therapy against *Campylobacter*, we investigated the distribution of the clustered regularly interspaced short palindromic repeats (CRISPR) systems in fluoroquinolone (FQ)-resistant *Campylobacter jejuni*. A total of 100 FQ-resistant *C. jejuni* strains from different sources were analyzed by PCR and DNA sequencing to determine resistance-conferring mutation in the *gyrA* gene and the presence of various CRISPR systems. All but one isolate harbored 1–5 point mutations in *gyrA,* and the most common mutation was the Thr86Ile change. Ninety-five isolates were positive with the CRISPR PCR, and spacer sequences were found in 86 of them. Among the 292 spacer sequences identified in this study, 204 shared 93–100% nucleotide homology to *Campylobacter* phage D10, 44 showed 100% homology to *Campylobacter* phage CP39, and 3 had 100% homology with *Campylobacter* phage CJIE4-5. The remaining 41 spacer sequences did not match with any phages in the database. Based on the results, it was inferred that the FQ-resistant *C. jejuni* isolates analyzed in this study were potentially resistant to *Campylobacter* phages D10, CP39, and CJIE4-5 as well as some unidentified phages. These phages should be excluded from cocktails of phages that may be utilized to treat FQ-resistant *Campylobacter*.

## 1. Introduction

*Campylobacter jejuni* causes bacterial gastroenteritis in humans worldwide and is responsible for an estimated 1.3 million cases of diarrhea each year in the United States [1,2]. Campylobacteriosis is typically a self-limiting condition, with symptoms usually resolving within a week, but antimicrobial therapy may be necessary in immune-compromised and elderly patients [3,4].

*Campylobacter* spp. have been reported to be resistant to antibiotics including fluoroquinolones, beta-lactams, macrolides, and aminoglycosides [4,5]. Fluoroquinolones (e.g., ciprofloxacin) and macrolides (e.g., azithromycin) are the primary antibiotics used for treatment in humans, and thus resistance to these classes of drugs in *Campylobacter* is a significant public health concern [6,7]. Whereas fluoroquinolone resistance develops easily, macrolide resistance in *Campylobacter* is a gradual process requiring prolonged exposure to the antibiotic [8]. In *Campylobacter*, quinolone antibiotics exert their effect via binding to and interfering with the function of the DNA gyrase enzyme (consisting of GyrA and GyrB subunits), resulting in DNA breaks and cell death [9]. DNA gyrase plays an essential role in DNA repair, recombination, transcription, and replication. Of note, *Campylobacter* does not encode topoisomerase IV (ParC/ParE), which is known to be another main target of fluoroquinolones in many other bacteria [4,10]. 

Resistance to fluoroquinolones in *Campylobacter* is primarily mediated by point mutations in the quinolone resistance-determining region (QRDR) of GyrA, which is located within the DNA binding site on the surface of DNA gyrase [11]. A number of different amino acid substitutions in the QRDR such as Thr86Ile, Asp90Asn, Thr86Lys, Thr86Ala, Thr86Val, and Asp90Tyr have been reported to be associated with fluoroquinolone resistance in *Campylobacter* species, of which Thr86Ile (encoded by the C257T mutation in *gyrA* gene) is the most frequent one leading to clinically relevant levels of antibiotic resistance [8,12]. Of note, no mutations in GyrB have been found to be linked to fluoroquinolone resistance so far in *Campylobacter* [13]. In addition, the multidrug efflux pump CmeABC is critical for the development of resistance to fluoroquinolones in *Campylobacter* [4,14].

The clustered regularly interspaced short palindromic repeats (CRISPR) is an adaptive immune system that protects prokaryotes against foreign genetic elements [15,16]. CRISPR loci typically consist of short and highly conserved repetitive DNA sequences (up to 100 repeats) interspaced by variable short sequences of equal lengths (called spacers), and an adjacent 6 to 20 genes encoding CRISPR-associated (cas) proteins [17,18]. The CRISPR-Cas system is divided into three types (I, II, and III), each of which is further subdivided depending on the number and structure of the *cas* genes [19,20,21]. The bacteria such as *C. jejuni*, *Neisseria meningitidis*, *Haemophilus influenzae*, and *Pasteurella multocida* have the type II CRISPR-Cas system comprising the *cas1*, *cas2*, and *cas9* genes [15]. The *cas9* gene, which encodes the main protein component of type II CRISPR-Cas systems and mediates both the CRISPR RNA (crRNA) processing and the intervention stages, engages in spacer acquisition and exhibits the lowest level of diversity in protein structures [22]. This CRISPR system recognizes foreign DNA by the RNA-guided endonuclease Cas9 along with crRNA and trans-activating crRNA (tracrRNA) [23], commonly known as crRNA:tracrRNA duplex or sgRNA that targets a foreign genetic element [24]. Whereas the involvement of the CRISPR system in helping bacteria to defend against foreign invaders (i.e., phages) is well established, its role in antimicrobial resistance remains somewhat controversial [25]. Studies have shown that although there was no notable relationship between the *cas* gene presence and the pools of plasmids, integrons, or antimicrobial resistance determinants in *E. coli* [26], significant reverse associations between the presence of the CRISPR-Cas system and occurrence of antibiotic resistance were found in enterococci [27,28]. On the other hand, the CRISPR-Cas system in *C. jejuni* has been reported to increase antimicrobial resistance via regulation of certain genes [25]. 

Previous studies reported that Type II CRISPR-Cas loci are interchangeable by horizontal gene transfer, not only among different species of the same genus [29] but also among taxonomically distant bacterial species, although the ecological parameters involved in this process were not further investigated [30]. However, despite the potential of horizontal gene transfer, Type II CRISPR-Cas systems, which have a small operon size and a low diversity in gene content, are suitable for comparative genomics and phylogenetics analysis in bacteria [31]. 

Therapeutic use of phages is an approach employed in control of many foodborne bacteria, including *Campylobacter,* in the food production chain [32,33,34]. Phage therapy is also considered an alternative treatment method in the fight against antibiotic-resistant bacterial strains [35]. For example, several infectious diseases caused by multidrug-resistant bacteria have been mitigated successfully with the aid of phage therapy [36]. In addition, CRISPR-Cas9 technology has increasingly been employed to revolutionize the biological research on many fronts in recent years [35]. For instance, it was used successfully to introduce point mutations, deletions, and insertions into the lactococcal phage p2 [37]. CRISPR-Cas9 was also used to inject a red fluorescent protein into the *Klebsiella* phage phiKpS2 with an efficiency of 87.5% [38]. To make phage therapy effective, it is necessary to determine the distribution of phage–immune (i.e., CRISPR) systems in fluoroquinolone (FQ)-resistant *C. jejuni* isolates. The main focus of this study was to investigate the CRISPR system in FQ-resistant *C. jejuni* because FQ resistance is highly prevalent and alternative treatment strategies such as phage therapy are urgently needed to combat FQ-resistant *C. jejuni*. Toward this goal, we investigated the CRISPR systems of FQ-resistant *C. jejuni* isolates derived from various sources (human, animal, and environment). The information may be used to identify naive phages (to which FQ-resistant *C. jejuni* is not immune) that can potentially be used for the treatment of FQ-resistant *Campylobacter* in future. 

## 2. Results

### 2.1. Antimicrobial Susceptibility of All Isolates

Susceptibility profiles of 100 isolates of *C. jejuni* recovered from cattle, broiler chicken, turkey, and sheep feces as well as retail chicken meat to nine antimicrobial drugs were determined. All of the isolates showed resistance to either ciprofloxacin or nalidixic acid (99 were resistant to ciprofloxacin and 96 were resistant to nalidixic acid). There were four isolates resistant to ciprofloxacin (a fluoroquinolone) only and one isolate resistant to nalidixic acid only. Thus, for simplicity, all isolates were referred to as FQ-resistant in this study even though one isolate was resistant to nalidixic acid (a quinolone—not fluoroquinolone—antibiotic) only. The complete minimum inhibitory concentration (MIC) results are shown in Table 1.

### 2.2. Resistance Mechanism of FQ-Resistant C. jejuni Isolates

**Figure 1:**
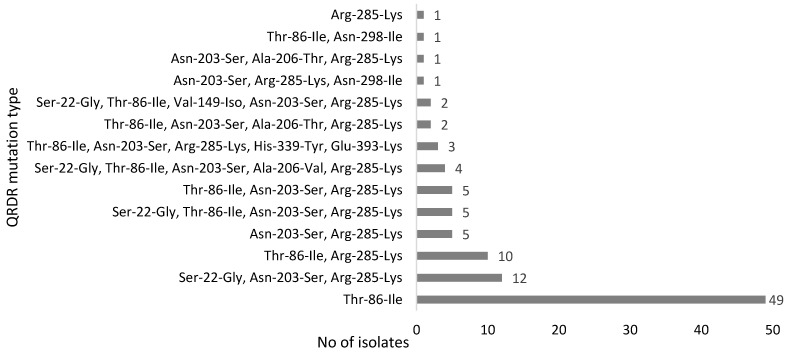
Point mutations observed in the quinolone resistance-determining region (QRDR) of GyrA in FQ-resistant *C. jejuni* isolates.

To examine the mechanisms of FQ resistance, the QRDR in *gyrA* of 100 *C. jejuni* isolates was sequenced to determine the mutations associated with FQ resistance. All of the isolates except for one harbored a single to five different types of mutations known to be associated with FQ-resistance in *Campylobacter*. The most common mutation was Thr-86-Ile. Fifty isolates had a single mutation, 16 isolates had two, 18 isolates had three, 7 isolates had four, and 9 isolates had five mutations. The phenotypic fluoroquinolone resistance observed in a single *C. jejuni* isolate that did not contain any mutations in the QRDR region of *gyrA* gene may have been due to other potential resistance mechanisms such as decreased outer membrane permeability and increased efflux activity [39]. Detailed information on the nonsynonymous mutation types of the isolates is show in Figure 1. In addition, synonymous (silent) mutations were detected frequently in the FQ-resistant *C. jejuni* isolates (results not shown). The most common silent mutations were C157T (*n* = 92), A186G (*n* = 50), G136A (*n* = 49), T119C (*n* = 46), and C81T (*n* = 38), and there was usually more than one mutation (mostly 4 to 10) in the QRDR region. The *gyrA* gene of *C. jejuni* NCTC 11,168 was used as a reference sequence for determining the mutations in the strains tested in this study.

### 2.3. CRISPR Detection, Spacer Identification, and Phylogenetic Analysis of C. jejuni Isolates

**Figure 2:**
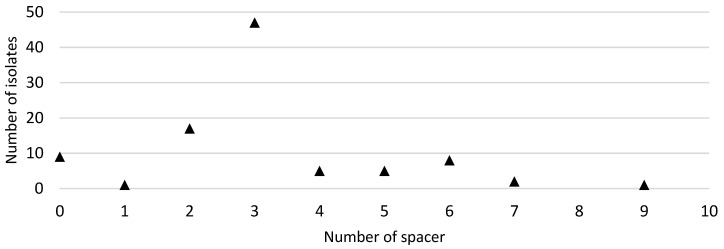
Distribution of the number of CRISPR (clustered regularly interspaced short palindromic repeats) spacers in 95 *C. jejuni* isolates from which CRISPR sequences were extracted by the CRISPR Recognition Tool.

**Figure 3:**
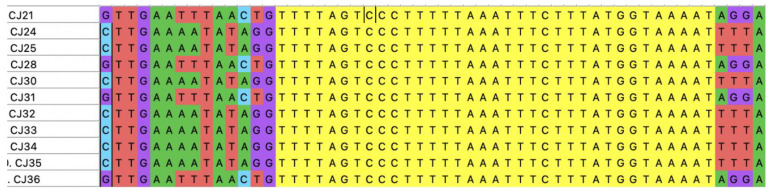
Representative CRISPR array sequences in FQ-resistant *C. jejuni* isolates examined in this study. CRISPR repeat regions (colored yellow) and various spacers surrounding the repeat regions on both sides are shown. The isolate names are depicted on the far left. The alignment was generated by MEGA X. The adenine (A), cytosine (C), guanine (G), and thymine (T), which are the letters of the DNA sequencing, showed as green, blue, purple, and red color, respectively.

In this study, the primer pair used targeted the conserved regions flanking the CRISPR-Cas locus in *C. jejuni*, which is located between the *moeA2* gene (cj1519) and a pseudogene (cj1528) in the genome [31]. Ninety-five out of the one-hundred FQ-resistant *C. jejuni* isolates were positive with the CRISPR array PCR. Spacer sequences were found in 86 out of the 95 CRISPR-positive isolates. The number of isolates and the number of spacers carried are shown in Figure 2. A total of 300 spacer sequences were determined and submitted to the NCBI database for sequence comparison via BLASTn. The distribution of lengths of spacer sequences was between 28 bp and 30 bp. Additionally, all of the 86 CRISPR spacer-positive FQ-resistant *C. jejuni* strains carried identical CRISPR repeat sequences of 35 bp in length between regions two and ten of the CRISPR locus (Figure 3).

The BLASTn analysis showed that the majority of the spacer sequences (*n* = 248) revealed a 93–100% homology to *Campylobacter* phage D10, while 44 sequences showed 100% homology with *Campylobacter* phage CP39. In addition, eight sequences had a 100% nucleotide homology with *Campylobacter* phage CJIE4-5. On the other hand, the remaining spacers (*n* = 9) did not match significantly with any *Campylobacter* or other phages available in the NCBI database. Detailed information on the sequencing results, including the annotation of spacers, is provided as supplementary data (Supplementary Table S1). 

**Figure 4:**
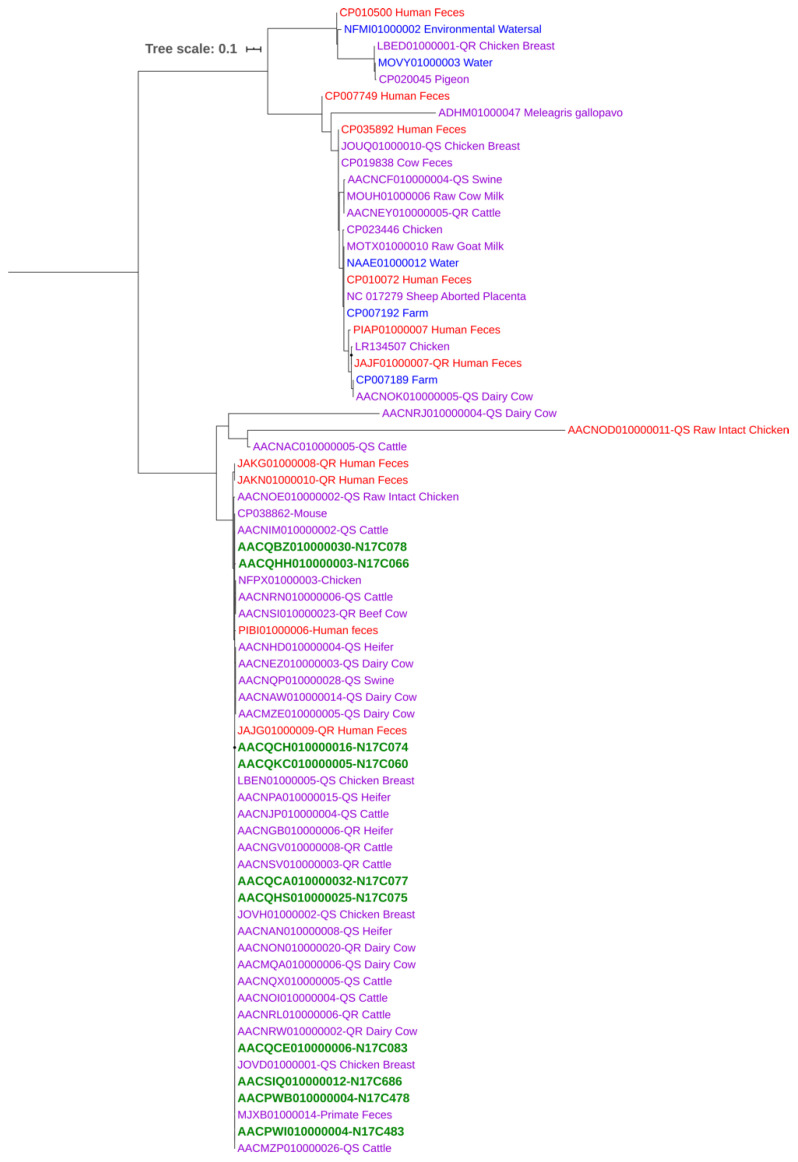
Cas9-based phylogenetic tree of *C. jejuni* isolates from different sources. Aligned sequences were used to construct a neighbor-joining tree with a bootstrap of 500 using MEGA X. Isolates are color-coded by their sources: Human (red), environmental (blue), animal (purple), and this study (green; all from chicken meat).

A phylogenetic tree based on the Cas9 protein amino acid sequences (inferred from the *cas9* gene sequences) of *C. jejuni* isolates from this study (*n* = 10; all from chicken meat) and those available at the PATRIC database (*n* = 59; from different sources) was generated with neighbor-joining estimation methods (Figure 4). The branching patterns demonstrated the presence of two main clades. The majority of the strains were clustered very closely in the same clade, which included animal, human, and all of the isolates from this study, indicating a close phylogenetic relationship based on Cas9. The smaller clade containing all the environmental isolates along with additional isolates from humans and animals displayed more divergence. The Cas9-based phylogenetic tree showed that *C. jejuni* isolates were overall clustered closely regardless of their isolation source, suggesting little or no relationship between Cas9 phylogeny and the origin of isolates and that the CRISPR-Cas system might disseminate readily among *C. jejuni* populations from diverse niches. 

## 3. Discussion

In this study, we first investigated the occurrence of CRISPR loci and spacers in FQ-resistant *C. jejuni* strains recovered from cattle, broiler, turkey, and sheep feces and retail chicken meat samples. In addition, phylogenetic relationships among *C. jejuni* isolates from various sources (including isolates from this study and those available at a public database) based on the Cas9 protein sequence were determined. Antimicrobial susceptibility testing confirmed that all of the isolates originated from this study were resistant to ciprofloxacin and/or nalidixic acid. The most common mutation in the *gyrA* gene was Thr-86-Ile.

The CRISPR-Cas system, which is encoded predominantly in the genomes of pathogenic bacteria that interact with eukaryotic hosts [40], was found to be present in the vast majority of the FQ-resistant *C. jejuni* isolates (95 out of 100) tested in this study, as determined by the PCR. All of these 95 isolates were shown to carry 35 bp long CRISPR repeat sequences (Figure 4). This same repeat sequence was also reported in *C. jejuni* in previous studies [41,42,43]. A previous study, which used greater than 4000 genome sequences to investigate the distribution of CRISPR-Cas in *C. jejuni* and *C. coli*, reported that 98.0% of *C. jejuni* strains were positive for CRISPR-Cas. On the other hand, a recent study showed that only 49% of the 99 *C. jejuni* isolates were positive for CRISPR-Cas [42]. Published work on the subject so far does not indicate the antimicrobial susceptibility profiles of *Campylobacter* isolates tested, but presumably contains both FQ-susceptible and FQ-resistant strains. Future studies are needed to better assess the distribution of the CRISPR locus in different populations of *C. jejuni*, including fluoroquinolone-susceptible vs. -resistant isolates. It should be noted that the mere presence of the CRISPR-Cas system in an organism does not necessarily indicate the functionality of the system [31]. The lack of functionality of the CRISPR-Cas system was reported to be related to the presence of chromosomally integrated mobile sialyltransferase containing loci and ganglioside-like lipooligosaccharide expression in *C. jejuni* [44]. 

We used the availability of CRISPR arrays [45] to search for potential targets of the FQ-resistant *C. jejuni* CRISPR-Cas system and found strong matches with *Campylobacter* phages D10, CP39, and CJIE4-5. *Campylobacter* phages are classified into three different groups (I, II, and III) based on the morphology and genome size [35,46,47]. In general, phages within a group share high genomic homology, but the overall homology among the different groups may be quite low [48]. Even though a few open reading frames (ORFs) of phage DA10 (a novel class of *Campylobacter* phage) have homologs in other *Campylobacter* phages, including CP39 (a class III *Campylobacter* phage) and the prophage CJIE4-5 [49], the likelihood of these shared ORFs in the induction of cross-immunity among different phage groups is expected to be minimal. Furthermore, these data showed that the CRISPR region of most of the FQ-resistant *C. jejuni* strains tested in this study contained nucleic acids derived not only from phages but also from plasmid or other sources. However, as with some arrays of CRISPR spacers, there were some spacers that did not match any plasmid/phage sequence in the NCBI database. As CRISPR arrays originate predominantly from genomes of mobile genetic elements, mostly viruses but also plasmids [50], this was not an unexpected finding.

The phylogenetic tree based on the Cas9 protein sequences showed that the *C. jejuni* isolates from humans were placed in the same cluster together with animal and environmental isolates of various origin (Figure 4). This finding indicated a close relationship between human, animal, and environmental isolates, suggesting that the CRISPR-Cas system may readily disseminate among *C. jejuni* strains from diverse isolation sources. The tree also indicated an overall moderate level of genetic diversity within the Cas9 protein sequences, suggesting the horizontal transfer of this gene among *C. jejuni* isolates with different genetic backgrounds. It has been reported that CRISPR spacers in *Staphylococcus* spp. can integrate with the mobile genetic element target sequences to facilitate a form of specialized transduction of CRISPR elements, indicating the role of CRISPR-Cas system in horizontal gene transfer [29]. More studies are required to understand the underlying mechanism of the transfer of the CRISPR-Cas system among the members of a bacterial species occupying different niches.

## 4. Material and Methods

### 4.1. Bacterial Culture and Identification

A total of 100 *C. jejuni* isolates from different sources (Table 2) were included in the current study. Pure culture (originated from a single colony grown on agar medium for 24 h) of each isolate was suspended in broth medium containing 30% glycerol and stored at −80 °C until use. From the frozen stocks, each strain was streaked onto Mueller–Hinton (MH) agar plate and incubated at 42 °C for 48 h under microaerobic conditions (5% O_2_, 10% CO_2_, and 85% N_2_) prior to use in this study. A single colony from each strain was subcultured onto a MH agar plate and incubated for 24 h under the same conditions for subsequent uses. 

Matrix-assisted laser desorption ionization–time-of-flight (MALDI-TOF) mass spectrometry (Bruker Daltonics, Billerica, Massachusetts, USA) was used for confirmation of all the isolates as *C. jejuni* included in this study. Sample preparation and analysis were done as described previously [54]. Mass spectra were acquired and analyzed using a microflex LT mass spectrometer (Bruker Daltonics) in combination with research-use-only version of the MALDI Biotyper Compass software 4.1 and the reference database MBT 7311 MSP Library (no. 1829023) at Iowa State University. Data were interpreted in accordance with the manufacturer’s (Bruker Daltonics) standard criteria, as follows: (i) high-confidence identification when the score was between 2.00 and 3.00, (ii) low-confidence identification when the score was between 1.70 and 1.99, and (iii) no organism identification possible when the score was 1.69 and lower.

### 4.2. Antimicrobial Susceptibility Testing

All of the 100 *C. jejuni* isolates (Table 2) were tested for their antimicrobial susceptibility profiles. The minimum inhibitory concentrations (MICs) of nine antibiotics were determined using a standard broth microdilution method as recommended by Clinical and Laboratory Standards Institute (CLSI) and the National Antimicrobial Resistance Monitoring System for Enteric Bacteria (NARMS). The tested ranges of the nine antibiotics are listed in Table 1. Commercially available Sensititre *Campylobacter* plates (Thermo Fisher Scientific, Waltham, MA, USA) were used for the test. The nine antibiotics were azithromycin, ciprofloxacin, erythromycin, gentamicin, tetracycline, florfenicol, nalidixic acid, telithromycin, and clindamycin. After incubation in a microaerobic environment for 24 h at 42 °C, the MICs were recorded and results were interpreted. For each isolate, the MIC value was set as the lowest antimicrobial concentration at which no bacterial growth was observed. The antimicrobial resistance breakpoints (Table 1) were chosen according to the standards established by NARMS and CLSI for bacteria isolated from animals [55,56,57]. *C. jejuni* ATTCC 33560 was included as the quality control strain for the MIC testing. 

### 4.3. PCR and Sequencing of gyrA for Mutation Determination

A total of 100 FQ-resistant *C. jejuni* isolates (as determined by the MIC test) were investigated for detection of the point mutations in *gyrA*. To amplify the QRDR region of *gyrA* by PCR, primers GyrAF1 (5′-CAACTGGTTCTAGCCTTTTG-3′) and GyrAR1 (5′-AATTTCACTCATAGCCTCACG-3′) were used [51,58]. All PCR products were purified using the QIAquick^®^ PCR purification kit (QIAGEN, Hilden, Germany) and then sequenced at the DNA Core Facility of Iowa State University using an Applied Biosystems 3730xl DNA Analyzer.

### 4.4. Detection of CRISPR Array and Analysis of CRISPR Spacers

The presence of the CRISPR-cas system in *C. jejuni* isolates was identified by conventional PCR as described previously [41]. Briefly, primers CRISPR-F (AGCTGCCCTTATGGTGGTG) and CRISPR-R (AAGCGGTTTTAGGGGATTGT) were used to identify the CRISPR region. The PCR reactions were performed in a 25 µL volume, containing 2 μL DNA template, 1 μL each primer (10 pmol), 2.5 µL 10× ExTaq™ buffer (TaKaRa, Shiga, Japan), 2 µL 2.5 mM each of deoxynucleotides triphosphate (dATP, dCTP, dGTP, and dTTP) and 0.5 µL ExTaq (TaKaRa, Shiga, Japan). The following touchdown PCR protocol was applied: denaturation at 95 °C for 30 s; primer annealing at 69 °C for 30 s; extension at 72 °C for 1 min, with lowering of the primer annealing temperature by 2 °C every 2 cycles until 59 °C was reached; and another 30 cycles with a primer-annealing temperature of 59 °C, followed by a final elongation step at 72 °C for 7 min. All PCR products were purified using the QIAquick^®^ PCR purification kit (QIAGEN) and then sequenced at the DNA Core Facility of Iowa State University using an Applied Biosystems 3730xl DNA Analyzer.

CRISPR spacer sequences were determined in all *C. jejuni* isolates that had a CRISPR array as identified by the PCR described above. The CRISPR Finder tool available online (http://crispr.u-psud.fr/Server/, accessed on 22 February 2020) was used to detect and identify CRISPR repeat and spacer sequences in the genome [45]. Similarities among sequences were searched in the BLASTn program (www.ncbi.nlm.nih.gov/BLAST/, accessed on 22 February 2020) against the GenBank nucleotide sequence database. Searches were conducted against all bacteria, and alignments having an E-value below the cut-off value with similarity greater than 80% were selected. Therefore, alignment search criteria were eventually based on sequence identity and E-value [25,59]. The repeat regions identified by the program were aligned using MEGA X [60] to evaluate their conservation.

### 4.5. Phylogenetic Analysis

The whole genome sequencing (WGS) data of retail chicken meat *C. jejuni* isolates (*n* = 10) from the current study (isolated as part of an ongoing NARMS surveillance project) were extracted from the NCBI Pathogen Detection Isolates Browser (https://www.ncbi.nlm.nih.gov/pathogens/isolates#/search/, accessed on 22 February 2020) for phylogenetic analysis.

Whole genome sequences of 59 *C. jejuni* (including both FQ-resistant and -susceptible strains) of animal (*n* = 44), human (*n* = 10), and environment (*n* = 5) origins at the PATRIC (https://patricbrc.org/, accessed on 22 February 2020) *C. jejuni* database were utilized for retrieving and inferring Cas9 protein sequences for phylogenetic analysis. GenBank accession numbers of genomes used in the phylogenetic comparison are shown in Figure 4. The Cas9-based tree was generated with neighbor-joining method with a bootstrap of 500 using the software MEGA X. The tree was visualized by the online tool iTOL (https://itol.embl.de/, accessed on 22 February 2020).

## 5. Conclusions

In summary, this study revealed the widespread presence of CRISPR-Cas systems in FQ-resistant *C. jejuni* isolates and identified bacteriophages to which *Campylobacter* is immune. These phages are potentially not effective against *C. jejuni* and should be excluded in the design of phage therapy. On the other hand, phages to which *Campylobacter* is not immune should be considered in the development of treatment regimes. This approach to phage therapy can be further facilitated by analyzing the *C. jejuni* genome sequences deposited in various databases.

## Figures and Tables

**Table 1:** Antimicrobial resistance profiles of 100 *C. jejuni* isolates tested in this study.

Antibiotic	Range (μg/mL)	Resistance Breakpoints (μg/mL)	* Sources (n)	No. of Isolates with an MIC (µg/mL) of:														No. (%) of Resistant Isolates
				0.015	0.03	0.06	0.12	0.25	0.5	1	2	4	8	16	32	64	>64	
AZ	0.015–64	≥8	CM (11)		9	2												0
			CF (70)		26	36	7	0	1									0
			BF (12)	1	10	1												0
			TF (4)		4													0
			SF (3)	1	2													0
CIP	0.015–64	≥4	CM (11)									2	3	5	1			11 (100.0)
			CF (70)									3	43	23	1			70 (100.0)
			BF (12)							1	0	4	7					11 (85.0)
			TF (4)									3	0	1				4 (100.0)
			SF (3)										1	2				3 (100.0)
ER	0.003–64	≥32	CM (11)				1	7	3									0
			CF (70)					22	38	6	2	1	1					0
			BF (12)				2	5	5									0
			TF (4)					3	1									0
			SF (3)					2	1									0
GN	0.12–32	≥8	CM (11)					1	9	1								0
			CF (70)			1	0	2	22	42	3							0
			BF (12)					4	8									0
			TF (4)					3	1									0
			SF (3)						3									0
TE	0.06–64	≥16	CM (11)				2	1	1	1	0	0	0	0	0	4	2	6 (55.0)
			CF (70)					1							1	4	64	69 (99.0)
			BF (12)				5	2							1	0	4	6 (46.1)
			TF (4)														4	4 (100.0)
			SF (3)													1	2	3 (100.0)
FL	0.03–64	≥16	CM (11)						1	10								0
			CF (70)						1	62	5	2						0
			BF (12)					4	6	2								0
			TF (4)						4									0
			SF (3)						1	1	1							0
NA	4.0–64	≥32	CM (11)												1	0	10	11 (100.0)
			CF (70)											1	1	4	64	69 (99.0)
			BF (12)														12	12 (92.3)
			TF (4)														4	4 (100.0)
			SF (3)									3						0
TEL	0.015–8	≥16	CM (11)					2	6	3								0
			CF (70)						5	51	11	3						0
			BF (12)							12								0
			TF (4)							4								0
			SF (3)						3									0
CL	0.03–16	≥8	CM (11)			2	9											0
			CF (70)			8	47	12	3									0
			BF (12)		2	3	7											0
			TF (4)			3	1											0
			SF (3)			1	1	1										0

* CM: chicken meat, CF: cattle feces, BF: broiler feces, TF: turkey feces, SF: sheep feces, AZ: azithromycin, CIP: ciprofloxacin, ER: erythromycin, GN: gentamicin, TE: tetracycline, FL: florfenicol, NA: nalidixic acid, TEL: telithromycin, CL: clindamycin.

**Table 2:** *Campylobacter jejuni* isolates used in this study.

Sources	No. Isolates	Origin
retail chicken meat	11	This study
cattle feces	10	Tang et al. [51]
cattle feces	60	This study
broiler feces	12	Luangtongkum et al. [52]
turkey feces	4	Luangtongkum et al. [52]
sheep feces	3	Xia et al. [53]
TOTAL	100	

## Data Availability

The sequence files supporting the findings of this article will be available in the NCBI GenBank database under accession numbers MW623446-MW623539 (CRISPR *cas9* gene) and MW623540-MW623629 (*C. jejuni gyrA* gene).

## Supplementary Materials

The following are available online at https://www.mdpi.com/2076-0817/10/3/345/s1: Table S1: Spacer sequences (alleles) of *Campylobacter jejuni* isolates in this study.
